# Investigating the Role of Psychological, Social, Religious and Ethical Determinants on Consumers’ Purchase Intention and Consumption of Convenience Food

**DOI:** 10.3390/foods10020237

**Published:** 2021-01-24

**Authors:** Hena Imtiyaz, Peeyush Soni, Vimolwan Yukongdi

**Affiliations:** 1 School of Environment, Resource and Development, Asian Institute of Technology, P.O. Box 4, Klong Luang, Pathumthani, Bangkok 12120, Thailand; st116566@ait.ac.th; 2 Agricultural and Food Engineering Department, Indian Institute of Technology Kharagpur, Kharagpur 721302, India; 3 School of Management, Asian Institute of Technology, P.O. Box 4, Klong Luang, Pathumthani, Bangkok 12120, Thailand; vyukongdi@ait.ac.th

**Keywords:** convenience food, determinants, purchase intention, consumption, confirmatory factor analysis, structural equation modeling

## Abstract

Despite impressive market growth, increasing demand and economic importance of convenience food in emerging economies such as India, comprehensive research regarding the role of psychological and social determinants on convenience food choice is lacking. Therefore, this research aims to investigate the influence of convenience orientation, social status, moral attitude, mood, spiritual concern, religious beliefs and ethical values on purchase intention and consumption of convenience food. The non-probability purposive sampling method was adopted for recruitment of participants. A pre-tested questionnaire was used to collect data from 501 consumers. The descriptive statistics, confirmatory factor analysis and structural equation modeling were carried out to analyse the data. The factor loading, Cronbach’s alpha, composite reliability, average variance extracted, and correlations demonstrated good internal consistency and reliability of scale items as well as convergent and discriminant validity of the constructs. The model fit indices revealed that measurement and structural models fitted well with data. The path analysis of the structural model demonstrated that convenience orientation (β = 0.789 ***, *t* = 32.462), moral attitude (β = 0.594 ***, *t* = 20.984), mood (β = 0.586 ***, *t* = 18.683), spiritual concern (β = 0.145 ***, *t* = 3.23), religious beliefs (β = 0.451 ***, *t* = 14.787) and ethical values (β = 0.497 ***, *t* = 16.678) were positively related with purchase intention and consumption of convenience food (*** Significant at *p* ≤ 0.01). The path analysis of structural model also indicated that social status was not linked with purchase intention and consumption of convenience food. The convenience orientation was the key determinant influencing purchase intention and consumption of convenience food.

## 1. Introduction

Busy and hectic lifestyles, increase in working population and urbanisation, increase in per capita and disposable incomes, the diminishing trend of cooking skills and motivation, rapid expansion of convenience food retail chains, significant improvements in food processing and packaging technologies and significant change in food related lifestyles have increased the demand and consumption of convenience food in both developed and emerging economies [1]. The global convenience food market is expected to grow at a CAGR of 4.49% during 2018–2025 and is anticipated to surpass a total revenue of USD 1126.8 billion in 2025. The European convenience food market is expected to grow at a CAGR of 4.2% during 2019–2024. The convenience food market in the Asia Pacific region is expected to grow at a CAGR of 8.79% during 2020–2025. The Indian convenience food market generated revenue of USD 261 million in 2017. It is predicated to grow at a CAGR of 16.24% during 2019–2024 and reach a revenue of USD 931 million in 2024 [1].

Convenience orientation is a key determinant influencing shopping for and consumption of convenience food in both developed and developing countries. The demand and consumption of convenience food are considerably increasing in emerging economies like India [2,3]. Several factors are favouring this trend, including a significant increase in women’s employment, diminishing trend of cooking skills and motivation, busy work schedule and considerable change in food habits. The majority of youth and dual working families prefer to spend minimal time on cooking and cleaning due to time scarcity induced by multiple factors, which drives them towards convenience food choice [4,5,6,7,8]. The role of convenience orientation on convenience food choice varies considerably with demographic, social, cultural and economic conditions [9,10,11].

Moral attitude is also an important determinant influencing shopping for and consumption of convenience food. The moral attitude in the context of shopping for and consumption of convenience food is influenced by moral obligation, moral and social norms, religious beliefs and ethical values [12,13]. Previous studies carried out in developed and industrialised nations reveal that moral attitude is one of the most important factors, which influences consumer perception, purchase intention and consumption of convenience food. The moral concerns towards health, safety and environmental issues have a significant influence on shopping for and consumption of convenience food [12,13,14]. Mood, which is a temporary state of mind, is another important determinant that drives consumers towards shopping for and consumption of convenience food [9,15]. Generally, the consumer perceives that convenience food reduces stress, minimises daily hassles, helps to deal with multiple responsibilities induced by a busy lifestyle and provides opportunities for leisure and social activities [10,16,17].

Spirituality is another important determinant, which influences consumers′ food choices. The dietary practices proposed and guided by spiritual norms may prevent food-related diseases. The influence of spirituality on shopping and eating behaviour of consumers varies significantly with religious beliefs, traditions, culture and socio–economic conditions. The food-related spiritual guidelines have a positive effect on human health as well as resulting in a better food-related lifestyle [18]. Religious belief is also an important determinant that influences the shopping and eating behaviour of consumers [19,20]. Religious beliefs and practices are positively associated with healthy food consumption as well as healthy lifestyle. The religious beliefs, rituals and values influence consumer psychology and behaviour towards purchase intention and consumption of convenience food [18,21,22]. In recent years, ethical values have emerged as one of the most important issues in the context of shopping for and consumption of convenience food. The ethical issues in the production and marketing of convenience food include farmer, labour and animal welfare; environment protection; social justice; human rights; place of origin and food certification. The ethical values in concerning shopping for and consumption of convenience food vary significantly with social, cultural, economic, religious and political differences as well as with consumer attitudes [23,24,25,26].

Socio–demographic trends in emerging economies like India have recently been indicating a major shift. These include a more educated and entrepreneurial youth population residing in megacities, an increased proportion of monthly income spent on food, lack of time to spend on cooking, multiple income families where almost every active person is employed, and above all the dynamic lifestyle [27]. The confluence of these driving vectors leads to a forthcoming sprawl of convenience food [3,28,29]. Several studies in the recent past have been carried out to seek the effect of various factors on convenience food choice, most of which focused on markets in developed and industrialised countries [8,10,12,13,14,15,16,22,26,30,31]. Due to diversity in tradition, culture, food habits, social structure, religious belief and ethical values, the consumers in emerging economies such as India, might not respond to such factors in the same way as reported in the aforementioned studies. Hence, it is important as well as timely to administer such research. Considering the impressive market growth and economic importance of convenience food in emerging economies such as India, the primary objective of the present study is to examine the association between convenience orientation, social status, moral attitude, mood, spiritual concern, religious belief and ethical values with consumer′s purchase intention and consumption of convenience food.

## 2. Theoretical Background and Development of Hypotheses

### 2.1. Convenience Orientation

Convenience orientation plays a significant and important role in motivating and driving consumers towards purchase intention and consumption of convenience food. Due to busy and hectic work schedules, a competitive environment, lack of cooking skills and motivation, desire for higher leisure time, multiple responsibilities, and significant change in food-related lifestyle, consumers in both developed and emerging economies seek convenience meal solutions, which in turn drive them towards purchase and consumption of convenience food [4,8,32,33]. Olsen et al. [2] revealed that convenience orientation was the key determinant influencing convenience food choice in Poland, followed by Spain and Netherlands. Januszewska et al. [10] indicated that convenience orientation, sensory appeal, health, price, and mood were the key determinants influencing convenience food choice in Belgium, Hungary, and Romania. In light of the aforementioned research findings, the following hypothesis is proposed.

**Hypothesis** **1** **(H1).** Convenience orientation has a positive influence on purchase intention of convenience food.

### 2.2. Social Status

Social status/social class is one of the important factors influencing consumers towards the purchase and consumption of convenience food. The diet of higher and lower socioeconomic status consumers differs significantly in terms of nutritive value, motives of food choice, taste, and variety of food [34,35]. Prattala et al. [36] stated that social status/social class has a significant influence on the food consumption attitude of the consumer. Mollen et al. [37] reported that the social environment plays an important and significant role in consumer food choice. Pechey and Monsivais [38] revealed that higher in-service social class was associated with shopping for and consumption of healthy food products. However, the role of social class/social status on purchase and consumption of convenience food is ambiguous. Considering the aforementioned research findings, the present study proposed the following hypothesis.

**Hypothesis** **2** **(H2).** Social status has a positive influence on purchase intention of convenience food.

### 2.3. Moral Attitude

Moral attitude is an important factor that influences convenience food choice. The convenience food production, packaging, storage, transportation, marketing and consumption are associated with moral and ethical issues. The moral attitude in the context of purchase and consumption of convenience food is influenced by social, cultural, religious and ethical norms and practices [14,30,39,40,41]. Olsen et al. [42] reported that moral obligation had a negative effect on purchase intention and consumption of ready to eat meals. They further stated that cultural differences in the context of moral attitude play a key role in the consumption of ready-to-eat meals in the Netherlands and Norway. Arbit et al. [13] stated that moral concern predicted moral engagement around the food product, which in turn predicts a sustainable eating pattern in USA and Germany. In light of the above-mentioned research findings, the following hypothesis is proposed.

**Hypothesis** **3** **(H3).** Moral attitude has a positive influence on purchase intention of convenience food.

### 2.4. Mood

Mood, whether positive or negative, is associated with shopping for and consumption of convenience food. It is generally perceived that convenience food helps to deal with multiple responsibilities induced by a busy lifestyle, which provides opportunities for relaxation and leisure activities [15,16]. The previous studies which were carried out mostly in developed and industrialised countries indicated that positive mood promotes healthy food consumption, whereas negative mood drives consumers towards unhealthy food consumption [10,15,16,43,44,45,46]. Based on the aforementioned research findings, the present study proposed the following hypothesis.

**Hypothesis** **4** **(H4).** Mood condition has a positive influence on purchase intention of convenience food.

### 2.5. Spiritual Concern

Spirituality largely directs food-related behaviour of consumers by condemning some foods and promoting others. Spirituality has a positive association with the well-being and health of consumers. Spirituality may encourage the consumer to purchase and consume healthy food and discourage unhealthy food [18]. The studies carried out in the past revealed that dietary practices proposed by spiritual norms and principles motivate and drive consumers towards healthy food consumption as well as better food related lifestyle [22,47]. On the contrary, some studies reported a negative association between spirituality, diet quality and health [18,48]. In light of the aforementioned research findings, the following hypothesis is proposed.

**Hypothesis** **5** **(H5).** Spiritual concern has a positive influence on purchase intention of convenience food.

### 2.6. Religious Beliefs

The shopping and eating behaviour of the consumer is also governed by religious, social, cultural and traditional factors. Food-related lifestyle and behaviour vary significantly amongst the followers of different religions [49]. Religious laws and practices prevent consumers from shopping for and consumption of a particular food product. Religious beliefs influence consumer psychology and behaviour towards shopping for and consumption of food. Religious beliefs and practices are positively associated with healthy food choices as well as a healthy lifestyle [19,20,21,22,50]. Suki and Suki [21] revealed that Muslim consumers were specific regarding shopping for and consumption of food products that comply with Islamic dietary laws and practices. Considering the above-mentioned research findings, the present study proposed the following hypothesis.

**Hypothesis** **6** **(H6).** Religious belief has a positive influence on purchase intention of convenience food.

### 2.7. Ethical Values

Ethical values have emerged as one of the most important issues in the context of convenience food choice. The major components of ethical issues are animal welfare, ecological welfare, planet protection, environmental protection, healthy eating, fair trade, labour welfare, and human rights concerning production, processing, packaging, transportation, storage and marketing of convenience food [23,24]. The previous studies revealed that ethical values play a significant and important role in convenience food choice [23,26]. On the contrary, Prescott et al. [9] and Januszewska et al. [10] revealed that ethical concern was the least important factor influencing convenience food choice in New Zealand, Taiwan, Malaysia, Hungary, Belgium, Philippines, and Romania. In light of the above-mentioned findings, the present study proposed the following hypothesis.

**Hypothesis** **7** **(H7).** Ethical values have a positive influence on the purchase intention of convenience food.

### 2.8. Purchase Intention and Consumption

The purchase intention of consumers towards convenience food is a complex process and is governed by a wide range of determinants. However, the importance of each determinant that drives consumers towards purchase intention of convenience food depends on food-related attitude and behaviour [26,33,51]. The perceived value of products which are directly, associated with convenience, sensory, quality, safety, health and price has a positive influence on consumers′ purchase intention for convenience food [52]. Apart from social, cultural, economic, religious and ethical factors, convenience food consumption is also influenced by convenience, sensory appeal, quality attributes, safety attributes, healthiness and price [17,53]. In light of the aforementioned research findings, the following hypothesis is proposed.

**Hypothesis** **8** **(H8).** Purchase intention is positively related to the consumption of convenience food.

**Figure 1:**
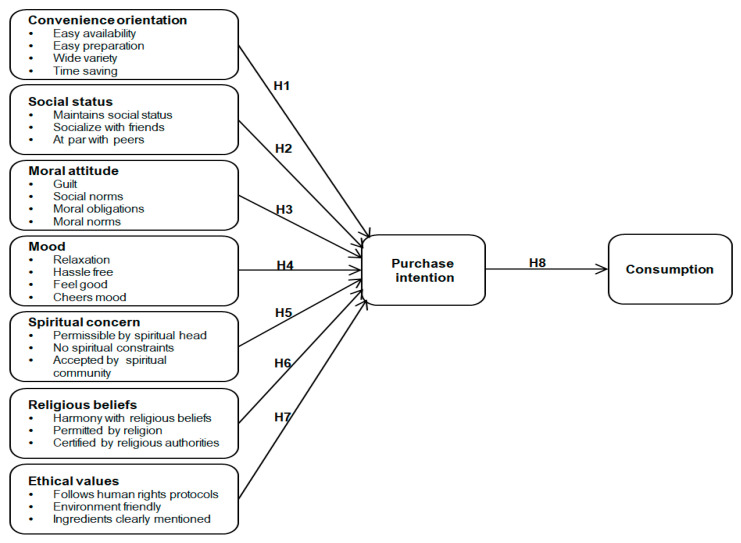
Conceptual model for convenience orientation, social status, moral attitude, mood, spiritual concern, religious beliefs and ethical values influencing purchase intention and consumption of convenience food.

The conceptual model for the current study is based on aforementioned research findings to assess the role of convenience orientation, social status, moral attitude, mood, spiritual concern, religious beliefs and ethical values influencing purchase intention and consumption of convenience food (Figure 1).

## 3. Materials and Methods

### 3.1. Development, Pre-Testing and Structure of the Questionnaire

Development of a questionnaire is crucial for the research study because oversight may lead to collection of irrelevant and inaccurate data [54,55]. The questionnaire was developed based on previous studies carried out in relation to convenience orientation, social status, moral attitude, mood, spiritual concern, religious beliefs and ethical values influencing convenience food choice as well as suggestions obtained from participants comprising of students, in-service personnel, food and nutrition experts and food technologists. A comprehensive literature review (Table 1) and feedback from participants, provided guidelines to develop the questionnaire to examine the role of the aforementioned determinants on purchase intention and consumption of convenience food. The pre-testing of the questionnaire was carried out at Sam Higginbottom University of Agriculture, Technology and Sciences, Allahabad, India to ensure the accuracy and reliability of the questionnaire [56]. The designed questionnaire was pre-tested with thirty participants as suggested by researchers [57,58,59]. The purposive sampling method was adopted to recruit the participants for the pre-testing of the questionnaire [18,33,60]. The participants comprised of university students and staff, professionals, food and nutrition experts and food technologists as they are the major consumers of convenience food. The participants were briefed about the purpose and objectives of the study as well as structure of the questionnaire. The participants were requested to identify and remove potential problems as well as to ensure its comprehensibility. After completing the questionnaire, the participants were asked to give their feedback about the design, structure and interpretation of the questionnaire to assess the role of aforementioned determinants on purchase intention and consumption of convenience food. The suggestions made by the participants were included in the final questionnaire to ensure accuracy and precision in data collection [60,61,62,63].

The questionnaire was divided into ten sections. The structure of the questionnaire was based on the proposed conceptual model relating convenience orientation, social status, moral attitude, mood, spiritual concern, religious beliefs and ethical values with purchase intention and consumption of convenience food. Section one of the questionnaire was constructed to collect general information from consumers i.e., educational qualification, occupation, marital status, food habits, preferences, frequency of eating convenience food and religious/ethnic background. The second section of the questionnaire was framed to collect information regarding the role of various aspects of convenience orientation on purchase intention of convenience food. The third, fourth, fifth, sixth, seventh and eighth sections of the questionnaire were designed to collect data about the various aspects of social status, moral attitude, mood, spiritual concern, religious beliefs and ethical values, respectively, on purchase intention of convenience food. The ninth and tenth sections of the questionnaire were planned to collect data for purchase intention and consumption of convenience food.

### 3.2. Participants

The participants were comprised of students and teaching and non-teaching staff from universities and colleges as well as professionals from banking, IT and real estate sectors. The participants consisted of 41.3% of males and 58.7% females. The age of the participants ranged from 18 to 65 years (average age = 30.37 years). The participants comprised of 48.9% single and 51.1% married in which 34.1% and 65.9% were unemployed and employed, respectively. The participants′ educational level ranged from high school to doctoral i.e., high school (0.40%), senior secondary school (7.0%), diploma (1.4%), undergraduate (33.9%), masters (34.5%), and doctoral (22.8%). The annual family income of the participants varied from INR 50,000 to INR 3,000,000 (Table 2).

### 3.3. Sampling Method and Sample Size

The non-probability purposive sampling method was adopted for the recruitment of the participants because researchers were targeting a specific group of participants as they are major consumers of convenience food products [18,33,60]. The present study was comprised of 550 participants from four major cities of northern India i.e., Allahabad, Lucknow, Noida and Aligarh. The total population of the four cities is approximately 8.25 million. The sample size of 550 participants taken in this study was more than 400 as recommended for a population over 0.25 million with a confidence level of 95% and 5% margins of error [60,67]. A total of 49 participants were eliminated because they provided incomplete information. Thus, the final sample size was 501, with a response rate of 91.09%.

### 3.4. Data Collection

The structured and pre-tested questionnaires were distributed to 550 participants in four universities, eight colleges and twelve corporate offices in January 2019. The participants were requested to gather at the conference/meeting rooms provided by the universities, colleges and corporate sectors. The participants were informed one day in advance regarding time and venue to achieve the desired number of participants as well as to avoid inconvenience. A group of 25 participants were invited to complete the questionnaire. The researcher distributed the questionnaire to the participants and briefed them about purpose, objectives and importance of the study. The influence of aforementioned determinants on purchase intention and consumption of convenience food were determined on five-point Likert scale (strongly disagree = 1, disagree = 2, don’t know = 3, agree = 4, strongly agree = 5). The participants were asked to choose one from 1 to 5 for each question [8,60,64].

### 3.5. Data Analysis

The statistical software SPSS version 24 was used to determine the mean, standard deviation skewness and kurtosis of each item of the aforementioned constructs. Furthermore, SPSS version 24 was employed to determine Cronbach’s alpha of constructs to ensure internal consistency and reliability of scale items of the questionnaire [60,68,69]. The AMOS software version 23 was employed for confirmatory factor analysis (CFA) and structural equation modelling (SEM). The CFA was carried out to estimate factor loading, composite reliability, average variance extracted and model fit indices. The composite reliability of the constructs of the questionnaire was determined to examine the reliability of scale items [60,61,63,68]. The standardised factor loading and average variance extracted were determined to assess the convergent validity of the constructs of the measurement model [6,62,63,68]. Correlations amongst the constructs and square root of average variance extracted were used to examine the discriminant validity of constructs of the measurement model [70]. The statistical indices such as the Comparative fit index (CFI), Tucker-Lewis index (TLI), Goodness of fit index (GFI), Root mean square error of approximation (RMSEA) and Standardised mean square residual (SRMR) were determined to examine the fit of measurement model [8,60,71].

The structural equation modelling approach was adopted to test the postulated hypotheses [62,63]. The structural model was constructed to examine the relationship between convenience orientation, social status, moral attitude, mood, spiritual concern, religious beliefs, ethical values and purchase intention as well as purchase intention with consumption of convenience food. The CFI, TLI, GFI, RMSEA, SRMR and χ^2^/df (Chi-square/degree of freedom) were determined to assess the fit of the structural model [60,63,69]. Furthermore, modification of indices was performed to improve the overall fit of the structural model [62]. The standardised estimate (path coefficient), standard error, *t*-value and *p*-value were used to test the hypotheses [6,60,63].

## 4. Results

### 4.1. Descriptive Statistics

Table 3 shows the mean score of constructs and different items of constructs i.e., convenience orientation, social status, moral attitude, mood, spiritual concern, religious beliefs, ethical values, purchase intention and consumption of convenience food. The mean participants score indicated that convenience orientation was the most important determinant influencing purchase intention and consumption of convenience food, followed by moral attitude, mood, ethical values, religious beliefs and spiritual concern [2,10]. “Easy to prepare”/”time saving/minimum physical efforts in cooking and cleanup” within convenience orientation; “don’t feel guilty” within moral attitude; “feel good/helps to relax” within mood; “no constraint for consumption” within spiritual concern; “not forbidden” within religious beliefs and “ingredients properly marked” within ethical values were the important factors influencing purchase intention and consumption of convenience food (Table 3). The skewness for different items of convenience orientation, social status, moral attitude, mood, spiritual concern, religious beliefs and ethical values ranged from −0.918 to 0.505, which were within the threshold values of −1 to 1 (Table 3). The kurtosis for different items of convenience orientation, social status, moral attitude, mood, spiritual concern, religious beliefs and ethical values ranged from −1.357 to 1.933, which were within the threshold values of −2 to 2 (Table 3). The skewness and kurtosis values obtained for different items of the aforementioned constructs indicated that data/participants’ scores were normally distributed [42,69].

### 4.2. Measurement Model

Table 3 presents factor loading, Cronbachs alpha (α), composite reliability (CR), and average variance extracted (AVE) for convenience orientation, social status, moral attitude, mood, spiritual concern, religious beliefs, ethical values, purchase intention and consumption of convenience food. The factor loading of the aforementioned constructs exceeded the minimum cut off point of 0.50, therefore all items were included for the interpretation of the factors influencing purchase intention and consumption of convenience food [8,10,15,63,68,71]. Cronbach’s alpha for convenience orientation, social status, moral attitude, mood, spiritual concern, religious beliefs, ethical values, purchase intention and consumption constructs ranged from 0.731 to 0.901, which exceeded the minimum acceptable value of 0.70 [60,61,68,69]. Composite reliability of convenience orientation, social status, moral attitude, mood, spiritual concern, religious beliefs, ethical values, purchase intention and consumption constructs varied from 0.899 to 0.990, which exceeded the recommended cut off value of 0.70 [60,63,68]. Cronbach’s alpha and composite reliability values obtained for different constructs revealed good internal consistency and reliability of scale items of the questionnaire [10,63,71]. The average variance extracted (AVE) for convenience orientation, social status, moral attitude, mood, spiritual concern, religious beliefs, ethical values, purchase intention and consumption constructs varied from 0.538 to 0.864, which exceeded the threshold value of 0.50 [8,60,70]. The factor loading higher than 0.50 and average variance extracted higher than 0.50, confirmed the convergent validity of the constructs [8,60,70,71]. The square root of AVE estimates (diagonal values) was higher than the correlation estimates amongst constructs (Table 4), which confirmed the discriminant validity of the constructs [60,63,70].

The Comparative fit index (CFI), Tucker-Lewis index (TLI), Goodness of fit index (GFI), Root mean square error of approximation (RMSEA) and Standardised mean square residual (SRMR) were used to assess the fit of measurement model relating convenience orientation, social status, moral attitude, mood, spiritual concern, religious beliefs and ethical values with purchase intention and consumption of convenience food. The CFI was 0.915 (≥0.90); TLI was 0.908 (≥0.90); GFI was 0.903 (≥0.90); RMSEA was 0.079 (≤0.08) and SRMR was 0.054 (≤0.08), which were within the recommended threshold values (Table 3). The values of the aforementioned indices confirmed a good fit of the measurement model with the data [26,60,63,72].

### 4.3. Structural Model

**Figure 2:**
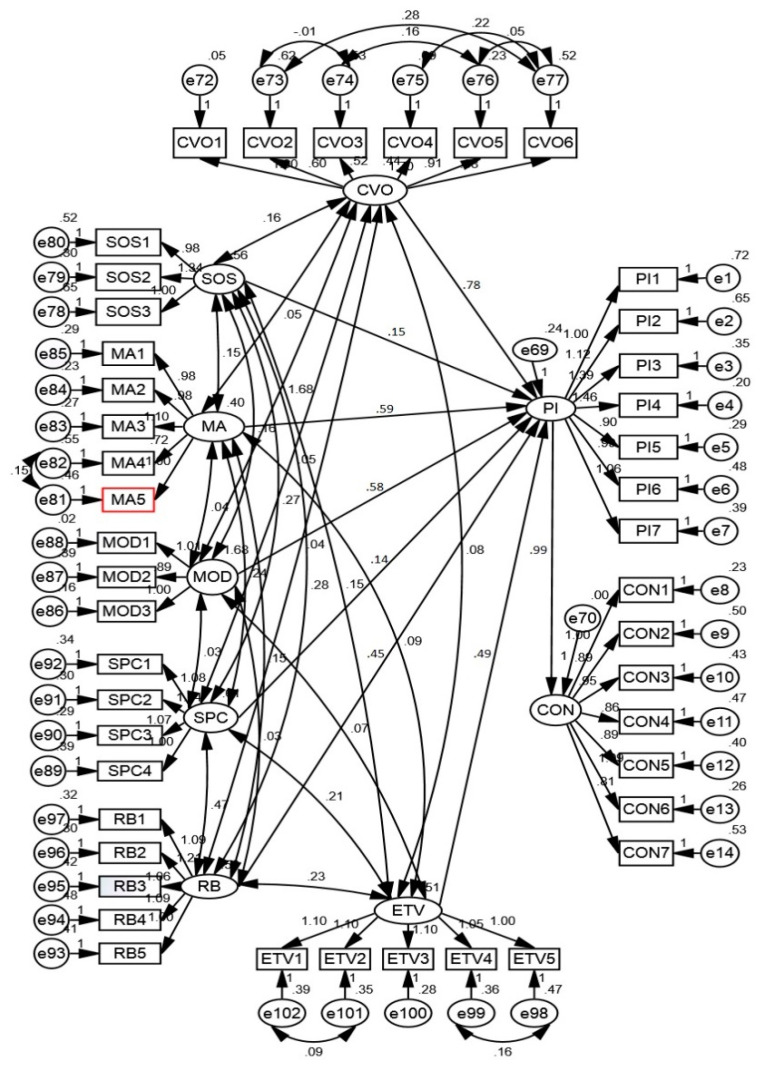
Structural equation modelling to assess the role of cultural and psychological determinants on purchase intention and consumption of convenience food. Structural model fit indices: CFI = 0.926; TLI = 0.908; GFI = 0.906; RMSEA = 0.077; SRMR = 0.068; χ^2^/df = 3.4. CVO: convenience orientation; SOS: social status; MA: moral attitude; MOD: mood; SPC: spiritual concern; RB: religious beliefs; ETV: ethical values; PI: purchase intention; CON: consumption.

The structural model was constructed to examine the association between convenience orientation, social status, moral attitude, mood, spiritual concern, religious beliefs, ethical values and purchase intention as well as purchase intention and consumption of convenience food. The CFI was 0.926 (≥0.90); TLI was 0.908 (≥0.90); GFI was 0.906 (≥0.90); RMSEA was 0.077 (≤0.08); SRMR was 0.068 (≤0.08) and χ^2^/df (chi square/degree of freedom) was 3.4 (<5.0), which falls within the recommended acceptable level (Figure 2). The aforementioned results demonstrated good fit of the structural model [60,63,69,72].

The results of structural model presented in Figure 2 and Table 5 demonstrated the extent of association between convenience orientation, social status, moral attitude, mood, spiritual concern, religious beliefs, ethical values and purchase intention as well as purchase intention with consumption of convenience food. Hypothesis 1 (H1), that postulated positive influence of convenience orientation on purchase intention of convenience food was supported as a standardised estimate (β) of the path of the structural model which was significant (β = 0.789, *t*-value = 32.462, *p* ≤ 0.01). Hypothesis 2 (H2), which proposed a positive influence of social status on purchase intention of convenience food was rejected because the standardised estimate of the path of the structural model was not statistically significant (β = 0.153, *t*-value = 1.484, *p* ≥ 0.230). Hypothesis 3 (H3), which predicted that moral attitude has a positive influence on purchase intention of convenience food was supported as the standardised estimate (β) of the path of the structural model was significant (β = 0.594, *t*-value = 20.984, *p* ≤ 0.01). Hypothesis 4 (H4) that proposed a positive influence of mood condition on purchase intention of convenience food was supported because the standardised estimate (β) of the path of the structural model was significant (β = 0.586, *t*-value = 18.683, *p* ≤ 0.01). Hypothesis 5 (H5) stated that spiritual concern would have a positive influence on purchase intention of convenience food was supported as the standardised estimate (β) of the path of the structural model was significant (β = 0.145, *t*-value = 3.23, *p* ≤ 0.01). Hypothesis 6 (H6) that proposed a positive influence of religious beliefs on purchase intention of convenience food was supported because the standardised estimate (β) of the path of the structural was significant (β =0.451, *t*-value = 1.787, *p* ≤ 0.01). The analysis of the structural model demonstrated a positive influence of ethical values on purchase intention of convenience food, supporting the Hypothesis 7 (H7), as the standardised estimate (β) of the path of the structural model was significant (β = 0.497, *t*-value = 6.678, *p* ≤ 0.01). Hypothesis 8 (H8), which postulated a positive influence of purchase intention on the consumption of convenience food was also supported because the standardised estimate (β) of the path of the structural model was significant (β = 0.998, *t*-value = 61.962, *p* ≤ 0.01). Furthermore, standardised estimate of the path of the structural model revealed that convenience orientation (β = 0.789), was the most important determinant, followed by moral attitude (β = 0.594), mood (β = 0.586), ethical values (β = 0.497), religious belief (β = 0.451) and spiritual concern (β = 0.145) influencing purchase intention and consumption of convenience food.

## 5. Discussion

Convenience orientation plays an important role in driving consumers towards convenience food consumption. The results of the structural model and mean participants score of the construct revealed that convenience orientation had a significant and positive influence on purchase intention and consumption of convenience food. The standardised estimate of the structural model indicated that convenience orientation was the most important factor influencing consumers for convenience food choice [2,10]. Furthermore, easy to prepare, time saving and minimum physical effort for preparation and clean up were the key factors which positively influenced purchase intention and consumption of convenience food [3]. Previous studies carried out under a wide range of social, cultural and economic conditions support the findings of the present study [5,9,31,64,73]. Olsen et al. [2] revealed that convenience orientation was the most important factor influencing convenience food consumption in Poland and Spain. Januszewska et al. [10] reported that convenience, sensory appeal, health, price and mood were the key determinants influencing convenience food choice in Belgium, Hungary and Romania. The food choice of consumers may vary with their social status/social environment. The analysis of the structural model indicated that social status had no significant influence on purchase intention and consumption of convenience food because the standardised estimate was statistically insignificant. Furthermore, the mean participant’s score of the construct as well as different items of the construct also revealed that social status had no significant effect on convenience food choice. The effect of social status/social class on convenience food choice is ambiguous [35,36]. However, Mollen et al. [37] reported that the social environment plays an important role in consumers’ food choice. They also stated that further study is required to gain in-depth knowledge regarding the role of social status/social environment on consumer food choice.

Moral attitude is an important factor that influences convenience food choice. The analysis of the structural model indicated that moral attitude had a significant and positive influence on purchase intention and consumption of convenience food. The mean participants’ score of construct and different items of construct revealed that moral attitude had a positive influence on purchase intention and consumption of convenience food. This is due to fact that consumers did not feel guilt, moral obligation, neglecting duties and destroying and promoting unhealthy food traditions during shopping for and consumption of convenience food. Furthermore, social and moral norms do not restrict the purchase and consumption of convenience food. On the contrary, the findings of other studies carried out in developed countries demonstrated a negative influence of moral attitude towards shopping for and consumption of convenience food [14,74]. Olsen et al. [12] reported that moral obligation had a negative effect on shopping for and consumption of ready to eat meals. They further stated that cultural differences in the context of moral attitude play a key role in the consumption of ready-to-eat meals in the Netherlands and Norway. Mood, whether positive or negative, is another important factor which influences purchase decision and consumption of convenience food. It is generally believed that convenience food reduces stress, minimises daily hassle, helps to cope with busy work schedule and provide opportunities for leisure activities [28,43]. The analysis of the structural model indicated that mood has a significant and positive influence on purchase intention and consumption of convenience food. Furthermore, the mean participants’ score of construct and different items within the construct also revealed that mood was an important motivating factor that positively influenced purchase intention and consumption of convenience food. The previous studies carried out to examine the role of mood on convenience food choice support the findings of the present research [10,15,16,17].

The influence of spiritual concern on purchase decision and consumption of convenience food may vary with religion, tradition, culture and socio-economic conditions [47]. The analysis of the structural model indicated that spiritual concern had a significant and positive influence on purchase intention and consumption of convenience food. Furthermore, the mean participants′ score of construct and different items within the construct revealed that spiritual concern had a positive influence on purchase intention and consumption of convenience food. This is due to fact that the spiritual norms do not restrict purchasing and consumption of convenience food in emerging economies like India. Tan et al. [18] reported that religion and spirituality had a significant influence on consumer food choice. They further revealed that a high level of spirituality might result in better food-related lifestyles. The religious laws and practices prevent the consumers from purchasing and consuming specific food products [21,22]. The analysis of the structural model indicated that religious beliefs had significant and positive influence on purchase intention and consumption of convenience food. Furthermore, the mean participants′ score of construct and different items within the constructs also revealed that religious beliefs had a positive influence on purchase intention and consumption of convenience food. This is due to fact that religious norms and guidelines do not restrict the purchase and consumption of convenience food. The results of the previous studies showed that religion/religious belief was an important determinant that influences consumers′ food-related attitude, behaviour and purchase decision [19,20,21].

In recent years, ethical values have become an important factor which influence purchase decision and consumption of convenience food [23,51]. The analysis of the structural model demonstrated that ethical values had a significant and positive influence on purchase intention and consumption of convenience food. Furthermore, the mean participant’s score of construct and different items within the construct also revealed that ethical values had positive influences on purchase intention and consumption of convenience food. The previous studies carried out under a wide range of social, cultural and economic conditions support the findings of this study [23,25,26]. On the contrary, Prescott et al. [9] reported that ethical value was the least important factor influencing consumers’ food choice in Taiwan, Malaysia, and New Zealand, but in Japan, ethical values made a significant contribution towards consumers’ food choice. Januszewska et al. [10] revealed that ethical values play an insignificant role in influencing consumers′ perception and consumption behaviour towards convenience food as compared to sensory appeal, convenience, price, health and mood in Hungary, Romania and the Philippines, but in Belgium, mood was found to be a less important factor than ethical values.

## 6. Conclusions

The findings of the present study highlight the role of convenience orientation, social status, moral attitude, mood, spiritual concerns, religious belief and ethical values on purchase intention and consumption of convenience food. The factor loading, average variance extracted and correlations indicated convergent and discriminant validity of constructs. The statistical indices demonstrated a good fit of measurement and structural models. Except for social status, convenience orientation, moral attitude, mood, spiritual concerns, religious belief and ethical values were positively associated with purchase intention and consumption of convenience food. The convenience orientation was found to be the most important motivating factor driving consumers towards convenience food choice mainly due to easy preparation, time-saving and minimum physical and mental effort in preparation/cooking, cleanup and waste disposal. The overall results indicate that moral, spiritual, religious and ethical norms and obligations do not restrict consumers for purchase and consumption of convenience food in developing and emerging economies such as India.

The conceptual framework and findings highlight some theoretical and practical contributions. Firstly, to the best of the author’s knowledge, this is the first comprehensive research carried out in emerging economies, particularly in India, to assess the role of aforementioned determinants on convenience food choice. Secondly, the empirical evidence indicates that, in emerging economies such as India, convenience orientation is becoming an important factor influencing consumers’ choice from traditional to convenience food. Thirdly, food processing industries and marketing agencies should note the paramount importance of spiritual, religious and ethical issues during production, processing, packaging and marketing of convenience food in order to serve their consumers better. Finally, food processing industries should obtain quality, safety, religious and ethical certifications from authorised agencies to enhance consumers’ trust in convenience food.

The present study has some limitations. Due to time and resource constraints, the present study was carried out in four cities in northern India, which limits the generalisation of the results. Hence, it is recommended to carry out similar research across cities and countries to obtain more generalisable and representative results.

## Figures and Tables

**Table 1:** Constructs of the questionnaire and their sources.

Constructs	Source
General information	Geeroms et al. [53]; Januszewska et al. [10]
Convenience orientation	Steptoe et al. [64]; Pula et al. [15]
Social status	Schultz et al. [35]; Mollen et al. [37]
Moral attitude	Olsen et al. [12]; Arbit et al. [13]; Hoek et al. [14]
Mood	Honkanen and Frewer, [16]; Loxton et al. [44]; Pula et al. [15];
Spiritual concern	Koieng et al. [47]; Tan et al. [18]; Mathras et al. [22];
Religious beliefs	Bakar et al. [19]; Aiedah, [20]; Mathras et al. [22]
Ethical values	Steptoe et al. [64]; Honkanen and Frewer, [16]; O′Connor et al. [26];
Purchase intention	Olsen et al. [12]; O′Connor et al. [26]; Ting et al. [33]
Consumption	Bae et al. [65]; Osman et al. [66]; Ting et al. [33]

**Table 2:** Socio–demographic profile of participants.

Socio-Demographic Variables	Groups	Number of Participants	Percentage of Participants
Gender	Male	207	41.32
	Female	294	58.68
Age (Years)	18–25	175	34.93
	26–35	203	40.52
	36–45	94	18.76
	46–65	29	5.79
Marital status	Single	245	48.90
	Married	256	51.10
Employment status	Unemployed	171	34.13
	Employed	330	65.87
Education level	10 *	2	0.40
	10 + 2 **	35	6.99
	Diploma	7	1.40
	Undergraduate	170	33.93
	Masters	173	34.53
	Doctoral	114	22.75
Annual family income (INR)	50,000–75,000	27	5.39
	75,000–100,000	32	6.39
	100,000–200,000	64	12.77
	200,000–500,000	140	27.94
	500,000–1,500,000	199	39.72
	1,500,000–3,000,000	37	7.39
	>3,000,000	2	0.40

Note: total sample size = 501; 1 USD = INR 72; * 10 = High School; ** 10 + 2 = Senior Secondary School.

**Table 3:** Mean participants’ score, factor loadings, Cronbach’s alpha (α), composite reliability (CR) and average variance extracted (AVE) of determinants influencing purchase intention and consumption of convenience food.

Construct	Items Code	Mean Score	Factor Loading	*p*-Value	α	CR	AVE
Convenience orientation (CVO)		4.21			0.731	0.990	0.9699
	CVO 1	3.60	0.978	***			
	CVO 2	3.51	0.923	***			
	CVO 3	3.98	0.729	***			
	CVO 4	3.65	0.984	***			
	CVO 5	4.22	0.721	***			
	CVO 6	4.21	0.665	***			
	CVO 7	4.21	0.686	***			
	CVO 8	3.72	0.953	***			
	CVO 9	3.76	0.919	***			
	CVO 10	3.91	0.790	***			
Social status (SOS)		2.79			0.890	0.899	0.582
	SOS 1	2.67	0.712	***			
	SOS 2	2.94	0.880	***			
	SOS 3	3.01	0.681	***			
Moral attitude (MA)		3.98			0.901	0.984	0.538
	MA 1	4.01	0.771	***			
	MA 2	3.92	0.774	***			
	MA 3	3.91	0.786	***			
	MA 4	3.83	0.801	***			
	MA 5	3.75	0.708	***			
	MA 6	3.71	0.713	***			
	MA 7	3.53	0.672	***			
	MA 8	3.80	0.717	***			
Mood (MOD)		3.79					
	MOD 1	3.59	0.985	***	0.765	0.961	0.768
	MOD 2	3.65	0.903	***			
	MOD 3	3.56	0.873	***			
	MOD 4	3.56	0.945	***			
	MOD 5	3.22	0.632	***			
Spiritual concern (SPC)		3.32			0.892	0.961	0.629
	SPC 1	2.90	0.658	***			
	SPC 2	3.31	0.829	***			
	SPC 3	3.24	0.847	***			
	SPC 4	3.18	0.827	***			
	SPC 5	3.03	0.791	***			
Religious beliefs (RB)		3.41			0.891	0.961	0.633
	RB 1	3.21	0.827	***			
	RB 2	3.46	0.853	***			
	RB 3	3.11	0.774	***			
	RB 4	3.30	0.759	***			
	RB 5	3.27	0.759	***			
Ethical values (ETV)		3.54			0.898	0.973	0.600
	ETV 1	2.94	0.664	***			
	ETV 2	3.22	0.814	***			
	ETV 3	3.12	0.824	***			
	ETV 4	3.32	0.794	***			
	ETV 5	3.52	0.789	***			
	ETV 6	3.45	0.753	***			
Purchase intention (PI)		4.21			0.780	0.900	0.576
	PI 1	4.14	0.628	***			
	PI 2	4.17	0.689	***			
	PI 3	3.65	0.842	***			
	PI 4	3.59	0.907	***			
	PI 5	3.50	0.754	***			
	PI 6	4.20	0.694	***			
	PI 7	3.93	0.763	***			
Consumption (CON)		3.95			0.740	0.940	0.690
	CON 1	3.83	0.900	***			
	CON 2	3.38	0.767	***			
	CON 3	3.79	0.826	***			
	CON 4	3.59	0.765	***			
	CON 5	3.81	0.816	***			
	CON 6	3.36	0.912	***			
	CON 7	3.67	0.741	***			

Measurement model fit indices: Comparative fit index (CFI) = 0.915; Tucker-Lewis index (TLI) = 0.908; Goodness of fit index (GFI) = 0.903; Root mean square error of approximation (RMSEA) = 0.079; Standardised mean square residual (SRMR) = 0.054; *** Significant at *p* ≤ 0.01; Skewness: −0.918 to 0.505; Kurtosis: −1.357 to 1.933. Note: See Appendix A for description of the items.

**Table 4:** Discriminant validity of the constructs.

Constructs	CVO	SOS	MA	MOD	SPC	RB	ETV	PI
CVO	0.984							
SOS	0.195	0.763						
MA	0.146	0.273	0.733					
MOD	0.413	0.172	0.101	0.876				
SPC	0.195	0.545	0.273	0.172	0.793			
RB	0.199	0.415	0.251	0.670	0.415	0.795		
ETV	0.155	0.249	0.219	0.228	0.249	0.422	0.774	
PI	0.369	0.105	0.294	0.540	0.405	0.374	0.674	0.759

**Table 5:** The structural model results examining the association of convenience orientation (CVO), social status (SOS), moral attitude (MA), mood (MOD), spiritual concern (SPC), religious beliefs (RB) and ethical values (ETV) influencing purchase intention (PI) and consumption (CON) of convenience food.

Hypothesis	Structural Path	Standardised Estimate (β)	Standard Error (SE)	*t*-Value	*p*-Value	Results
H1	CVO → PI	0.789	0.024	32.462	***	Supported
H2	SOS → PI	0.153	0.040	1.484	0.230	Rejected
H3	MA → PI	0.594	0.028	20.984	***	Supported
H4	MOD → PI	0.586	0.028	18.683	***	Supported
H5	SPC → PI	0.145	0.044	3.23	***	Supported
H6	RB → PI	0.451	0.031	14.787	***	Supported
H7	ETV → PI	0.497	0.032	16.678	***	Supported
H8	PI → CON	0.998	0.016	61.962	***	Supported

*** Significant at *p* ≤ 0.01.

## Appendix A

The description of the items code of the questionnaire.

**Convenience orientation (CVO)**
CVO 1—I prefer convenience food due to availability of variety of convenience food near to my residence
CVO 2—I prefer convenience food due to availability of variety of convenience food near to my workplace.
CVO 3—I prefer convenience food because it is easily available in supermarkets, grocery store and 24 h food outlets.
CVO 4—I prefer convenience food because it is easy to plan meals for family/guests with short notice.
CVO 5—I prefer convenience food because it is easy to prepare/cook.
CVO 6—I prefer convenience food because it requires little time to cook/prepare.
CVO 7—I prefer convenience food because it requires little physical effort to cook and clean up.
CVO 8—I prefer convenience food because it is easy to store.
CVO 9—I prefer convenience food because its waste disposal is easy.
CVO 10—I prefer convenience food because it makes life easier.
**Social status (SOS)**
SOS 1—I prefer convenience food in order to maintain my social status.
SOS 2—I prefer convenience food because it keeps me at par with my peers in the social circle.
SOS 3—I prefer convenience food because it gives me an opportunity to socialize with my friends.
**Moral Attitude (MOD)**
MA 1—I prefer convenience food because I don’t feel guilty on eating it.
MA 2—I prefer convenience food because I don’t feel any moral obligation restricting its purchase and consumption.
MA 3—I prefer convenience food because I don’t have any social norms restricting its purchase and consumption.
MA 4—I prefer convenience food because I don’t have any moral norms restricting its purchase and consumption.
MA 5—I prefer convenience food because I don’t feel that I am neglecting my duty for cooking from scratch
MA 6—I prefer convenience food because I don’t feel it will destroy my food tradition
MA 7—I prefer convenience food because I don’t feel it will make me lazy
MA 8—I prefer convenience food because I don’t feel I am promoting unhealthy food tradition
**Mood (MOD)**
MOD 1—I prefer convenience food because it helps me to relax.
MOD 2—I prefer convenience food because it makes me feel good.
MOD 3—I prefer convenience food because it helps me to cope with life.
MOD 4—I prefer convenience food because it minimizes my daily hassles.
MOD 5—I prefer convenience food because it cheers the mood of my spouse/children/friends/guests.
**Spiritual concern (SPC)**
SPC 1—I prefer convenience food because my spiritual head allows me to consume convenience food.
SPC 2—I prefer convenience food because as per my spiritual beliefs, I don′t have any constraints to consume convenience food.
SPC 3—I prefer convenience food because it is not forbidden in my spiritual community.
SPC 4—I prefer convenience food because it doesn’t constraint me to get involved in spiritual activities.
SPC 5—I prefer convenience food because it is certified by my spiritual community.
**Religious beliefs (RB)**
RB 1—I prefer convenience food because it is in harmony with my religion.
RB 2—I prefer convenience food because it is not forbidden in my religion.
RB 3—I prefer convenience food because it is certified by the religious authorities.
RB 4—I prefer convenience food because it is not mandatory to cook food from scratch in my religion.
RB 5—I prefer convenience food because it is permitted in religious functions.
**Ethical values (ETV)**
ETV 1—I prefer convenience food because it comes from friendly countries.
ETV 2—I prefer convenience food because it comes from a country which follows international standards for processing and packaging.
ETV 3—I prefer convenience food because it comes from countries following international human rights.
ETV 4—I prefer convenience food because it is produced and packed with environmentally friendly technologies.
ETV 5—I prefer convenience food because all the ingredients are properly marked on its packet.
ETV 6—I prefer convenience food because country of origin is clearly mentioned on its packets.
**Purchase intention (PI)**
PI 1—I will continue to buy convenience food due to competitive price and promotional offer.
PI 2—I will continue to buy convenience food to save time.
PI 3—I will continue to buy convenience food due to lack of cooking skills and motivation.
PI 4—I will continue to buy convenience food to reduce environmental damage.
PI 5—I will continue to buy convenience food due to good quality, safety and health.
PI 6—I will continue buy convenience food because it is readily available and easy to prepare.
PI 7—I will continue to buy convenience food as there are choices available for multi cuisines.
**Consumption (CON)**
CON 1—I consume convenience food due to convenience.
CON 2—I consume convenience food due to minimum physical and mental effort to cook.
CON 3—I consume convenience food due to good taste, smell and appearance.
CON 4—I consume convenience food due to attractive packaging.
CON 5—I consume convenience food due to competitive price.
CON 6—I consume convenience food due to good quality, high safety and healthiness.
CON 7—I consume convenience food due to my religious and ethical beliefs.
